# *setd2* knockout zebrafish is viable and fertile: differential and developmental stress-related requirements for Setd2 and histone H3K36 trimethylation in different vertebrate animals

**DOI:** 10.1038/s41421-020-00203-8

**Published:** 2020-10-20

**Authors:** Dian-Jia Liu, Fan Zhang, Yi Chen, Yi Jin, Yuan-Liang Zhang, Shu-Bei Chen, Yin-Yin Xie, Qiu-Hua Huang, Wei-Li Zhao, Lan Wang, Peng-Fei Xu, Zhu Chen, Sai-Juan Chen, Bing Li, Aijun Zhang, Xiao-Jian Sun

**Affiliations:** 1 Shanghai Institute of Hematology, State Key Laboratory of Medical Genomics, National Research Center for Translational Medicine at Shanghai, Ruijin Hospital Affiliated to Shanghai Jiao Tong University School of Medicine, Shanghai 200025, China; 2 CAS Key Laboratory of Tissue Microenvironment and Tumor, Shanghai Institute of Nutrition and Health, Shanghai Institutes for Biological Sciences, University of Chinese Academy of Sciences, Chinese Academy of Sciences, Shanghai 200031, China; 3 School of Life Sciences & Biotechnology, Shanghai Jiao Tong University, Shanghai 200240, China; 4 Division of Human Reproduction and Developmental Genetics, Women’s Hospital, and Institute of Genetics and Department of Genetics, Zhejiang University School of Medicine, Hangzhou, Zhejiang 310058 China; 5 Department of Biochemistry and Molecular Cell Biology, Shanghai Jiao Tong University School of Medicine, Shanghai 200025, China; 6 Reproductive Medical Center, Ruijin Hospital Affiliated to Shanghai Jiao Tong University School of Medicine, Shanghai 200025, China

**Keywords:** Epigenetics, Developmental biology

## Abstract

Setd2 is the only enzyme that catalyzes histone H3 lysine 36 trimethylation (H3K36me3) on virtually all actively transcribed protein-coding genes, and this mechanism is evolutionarily conserved from yeast to human. Despite this widespread and conserved activity, Setd2 and H3K36me3 are dispensable for normal growth of yeast but are absolutely required for mammalian embryogenesis, such as oocyte maturation and embryonic vasculogenesis in mice, raising a question of how the functional requirements of Setd2 in specific developmental stages have emerged through evolution. Here, we explored this issue by studying the essentiality and function of Setd2 in zebrafish. Surprisingly, the *setd2*-null zebrafish are viable and fertile. They show Mendelian birth ratio and normal embryogenesis without vascular defect as seen in mice; however, they have a small body size phenotype attributed to insufficient energy metabolism and protein synthesis, which is reversable in a nutrition-dependent manner. Unlike the sterile *Setd2*-null mice, the *setd2*-null zebrafish can produce functional sperms and oocytes. Nonetheless, related to the requirement of maternal *Setd2* for oocyte maturation in mice, the second generation of *setd2*-null zebrafish that carry no maternal *setd2* show decreased survival rate and a developmental delay at maternal-to-zygotic transition. Taken together, these results indicate that, while the phenotypes of the *setd2*-null zebrafish and mice are apparently different, they are matched in parallel as the underlying mechanisms are evolutionarily conserved. Thus, the differential requirements of Setd2 may reflect distinct viability thresholds that associate with intrinsic and/or extrinsic stresses experienced by the organism through development, and these epigenetic regulatory mechanisms may serve as a reserved source supporting the evolution of life from simplicity to complexity.

## Introduction

While the life’s evolution from simplicity to complexity can benefit living beings on survival and reproduction, this transition also put them under tremendous pressures and stresses, such as demanded requirements of nutrient redistribution between cells and sexual propagation^1,2^. To effect this evolutionary transition, living beings must cope with these extra stresses by means of (i) acquiring mutations (including creation of new genes and regulatory elements) to increase genomic complexity^1^, and/or (ii) exploiting the potential of epigenetic programs which enables the organism to regulate gene expression more diversely and precisely^2,3^. Potentially relevant to the latter strategy is the notable enigma that some highly conserved and widespread epigenetic mechanisms, such as the methylation of histone H3 lysine 4 (H3K4) and lysine 36 (H3K36), are not essential for some lower species (e.g., yeast) growing in normal conditions, but they are absolutely required for the viability of higher organisms including mammalians^4–7^. It is thus conceivable that some epigenetic mechanisms, though paradoxically “non-essential”, are important for the organisms to survive some stress conditions, which provides a basis to support the evolution of life from simplicity to complexity.

H3K36 tri-methylation (H3K36me3) represents one of the most conserved epigenetic marks through the evolution of eukaryotes^8^, and it is associated with virtually all actively transcribed protein-coding genes^9,10^. An evolutionarily conserved histone methyltransferase that catalyzes H3K36me3 is responsible for establishing this association between H3K36me3 and the active genes, as it directly and specifically interacts with the elongating form of RNA polymerase II^11–15^. The yeast and mammalian version of this histone methyltransferase, named Set2 and SETD2, respectively, share a similar domain architecture containing the catalytic AWS-SET-PostSET domains and the Set2 RNA polymerase II-interacting (SRI) domain^15,16^. Both Setd2 and SETD2 have been shown to be the sole enzyme responsible for H3K36me3 in their hosts^17–19^, although in higher organisms there emerge several less conserved SET domain family members that likely catalyze H3K36me1 and H3K36me2, whose genomic distribution patterns do not resemble that of H3K36me3^20^. The functions of Set2/SETD2 and H3K36me3 have been implicated in various aspects of gene regulation, including transcriptional elongation^11–14,21,22^, suppression of intragenic cryptic transcription^23,24^, nucleosome dynamics^25^, DNA repair^26^, DNA methylation^27,28^, *N*^6^-methyladenosine (m^6^A) mRNA modification^29^, and alternative mRNA splicing^30^. Furthermore, recent human cancer genomic studies have shown that somatic mutations, deletions, and dysregulated expression of SETD2, as well as altered H3K36me3 levels, are frequently identified in many types of cancers^31–35^, suggesting their pivotal roles in tumorigenesis and as potential therapeutic targets.

Despite such a high evolutionary conservation and the widely utilized functions of Set2/SETD2 and H3K36me3, loss of Set2 has little effect on the viability of yeasts in normal growth conditions; however, the *set2*-null yeast does show a slower growth in the condition of nutrition deprivation, compared with the wild-type^36,37^. In contrast, SETD2 is absolutely required for the viability and fertility of mammals, as a constitutive knockout of Setd2 in mice causes embryonic lethality due to defects in blood vessel development^19^, and Setd2 is also essential for spermiogenesis and oocyte development in mice^38,39^. Other tissue-specific studies in mice indicate that Setd2 regulates hematopoietic stem cell^40,41^ and bone marrow mesenchymal stem cell functions^42^, V(D)J recombination in lymphocytes^43,44^, as well as endodermal differentiation of embryonic stem cells^45^, though these functions may not necessarily be related to viability of the mice. These observations thus raise a question of how the functional requirements of H3K36me3 and Set2/SETD2 in the specific developmental stages have emerged through the evolution of life from simplicity to complexity. Here, we further explored this issue by using another vertebrate animal model, zebrafish, to investigate the essentiality and function of SETD2 in the development. It is surprising that *setd2* knockout zebrafish is viable and fertile. However, a close comparison between these model organisms suggests that the apparently differential requirements of H3K36me3 and Set2/SETD2 in different organisms are actually well paralleled and likely associated with intrinsic and/or extrinsic stresses experienced by the organisms through development.

## Results

### *setd2*-null zebrafish is viable

**Fig. 1:**
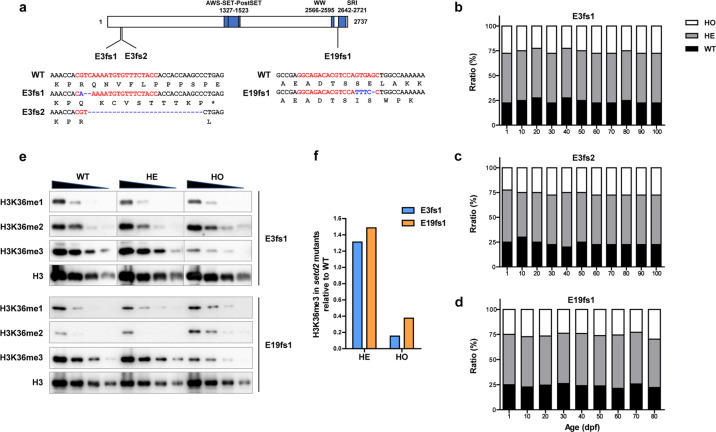
Zebrafish models with *setd2* loss-of-function mutations are viable. **a** Schematic diagram showing domain architecture of zebrafish Setd2 protein and mutated sites of the three mutant lines generated by CRISPR/Cas9 genome editing. The wild-type (WT) *setd2* mRNA is referred to the NCBI Reference Sequence XM_009292184.3. Two mutants contain frame-shift mutations in exon 3 and are therefore named E3fs1 (c.215G>A216_217del; p.R72Qfs*9) and E3fs2 (c.217_244del; p.Q73fs*133), both of which disrupt the majority of the protein. The third mutant contains a frame-shift in exon 19, named E19fs1 (c.7910_7913GTGA>TTTC7914del; p.S2637Ifs*34), which disrupts the C-terminal SRI domain. Genomic sequences and deduced amino acids around targeting sequences in *setd2* are shown. Red letters denote sgRNA-targeting sequences, while blue letters and dotted lines represent mutant sequences. **b–d** Genotype ratios of the WT, heterozygous (HE) and homozygous (HO) progenies, which are produced by self-cross of the three mutants, and their persistence through growth into adult. Note that they all fit the Mendelian Ratio. **e** Immunoblot analysis of the different H3K36 methylation states in the WT, HE and HO embryos of the E3fs1 and E19fs1 mutants. Total H3 was used as a loading control. Embryos at 72 hpf were analyzed. Each sample was loaded in 2-fold serial dilution to facilitate quantification. The immunoblot of the E3fs1 samples was actually analyzed in the same gels/explorations, but the images were rearranged just to fit the loading order in the lower panel. **f** Quantification of H3K36me3 levels in HE and HO relative to WT embryos of the E3fs1 and E19fs1 mutants.

To disrupt the function of Setd2 in zebrafish, we designed CRISPR/Cas9 genome editing strategies to target the N-terminal end (mutation within exon 3) or the C-terminal SRI domain (mutation within exon 19) of zebrafish Setd2 protein (Fig. 1a). As a result, three mutant lines were generated. Sequencing results validated that two of them carried frame-shift mutations in exon 3 and therefore were named as E3fs1 and E3fs2, whereas the third carried a frame-shift mutation in exon 19 and was named as E19fs1 (Fig. 1a). We performed RT-qPCR to analyze *setd2* mRNA levels in the embryos of the E3fs1 and E19fs1 lines, and the results showed a dramatic decrease of *setd2* mRNA in the homozygous mutants of E3fs1 but not E19fs1 (Supplementary Fig. S1). These results suggest that the N-terminal frame-shift mutation (in exon 3) could induce nonsense-mediated mRNA decay (NMD), whereas the C-terminal frame-shift mutation (in exon 19) could not, probably due to the short distance between the frame-shit site and the natural stop codon. Therefore, the E19fs1 mutation likely gives rise to a truncated Setd2 without SRI domain, so that the E3fs1 and E19fs1 mutants represent different models in which Setd2 is either completely depleted or inhibited by disrupting the Setd2-RNA pol II interaction.

We then analyzed the viability of the *setd2* mutant zebrafish. In contrast to our previous finding that the homozygous *Setd2* knockout mice are embryonic lethal^19^, it is surprising that self-cross of the heterozygous *setd2* mutant zebrafish, for each of the three mutant lines, could produce wild-type, heterozygous and homozygous embryos, and their genotype frequencies perfectly fit the Mendelian ratio (Fig. 1b–d). Furthermore, a follow-up genotyping of each mutant line indicated that this ratio was maintained through the growth from birth to adult (Fig. 1b–d), suggesting that Setd2 is dispensable for embryonic development and growth of zebrafish.

Of special note, a phenomenon called genetic compensation has recently been proposed to explain some very mild or no phenotypes in gene knockout animal models, especially compared with the corresponding gene knockdown models^46^. An important mechanism underlying this phenomenon is transcriptional activation of homologous genes by an NMD-initiated, RNA fragment-mediated recruitment of the histone H3K4-methylating enzymes (e.g., the COMPASS complex) to the promoters of the homologous genes^47,48^. To test whether this mechanism is relevant to our *setd2* mutant zebrafish, we first performed a Morpholino knockdown of Setd2. The results showed that, while the efficiency of Setd2 knockdown was verified by the significant inhibition of the EGFP reporter and the dramatic decrease of H3K36me3 (Supplementary Fig. S2a–c), the morphant embryos developed and grew normally (Supplementary Fig. S2b, d). We then performed RT-qPCR to examine whether the homologous genes of *setd2* was upregulated in the E3fs1 mutant, which exhibited NMD as described above. The results showed that the mRNA levels of the *setd2* homologues, including *ash1l*, *nsd1a*, *nsd1b*, *nsd2* (*whsc1*) and *nsd3* (*whsc1l1*), were not changed in the homozygous and heterozygous E3fs1 embryos, compared with the wild-type (Supplementary Fig. S3). Thus, these results suggest that the genetic compensation mechanisms may not be applicable to our observation of the non-essentiality of Setd2 in zebrafish viability.

Next, to determine the H3K36 methylation levels in the *setd2* mutant zebrafish, we performed immunoblot analysis of the embryos at 72 hour post fertilization (hpf) with methylation state-specific antibodies. The results showed that H3K36me3 was dramatically decreased in both E3fs1 and E19fs1 homozygous mutants, compared with the wild-type and heterozygous, whereas their H3K36me1 and H3K36me2 remained intact (Fig. 1e, f). These patterns are exactly the same as what we previously observed in the *Setd2* knockout mouse embryos^19^. The loss of H3K36me3 provides another line of evidence to exclude the genetic compensation effect and, in combination with previous studies, these results demonstrate that, while the specific role of Setd2 as the major enzyme for H3K36me3 is evolutionarily conserved, the functions of Setd2 and H3K36me3 are differentially required for the viability of different vertebrate animals.

### Unlike mouse, zebrafish vascular development is not impaired by loss of *setd2*

**Fig. 2:**
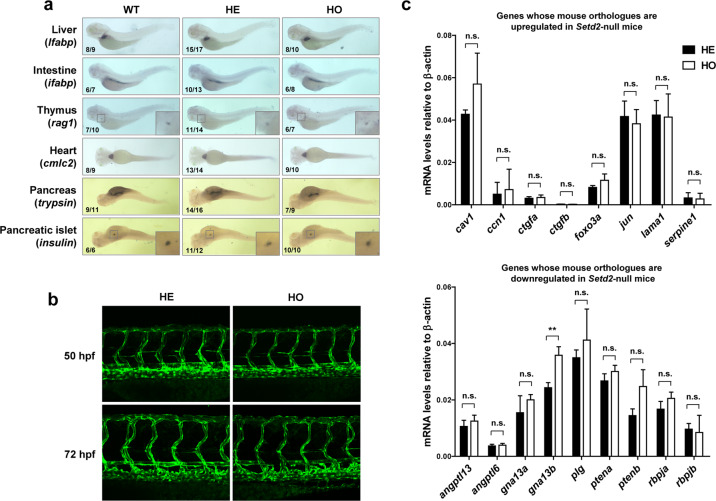
Normal organogenesis and vascular development in the *setd2*-null zebrafish. **a** Normal development of the major organs as indicated by whole mount in situ mRNA hybridization with specific markers (written in parentheses) in the embryos of indicated genotypes at 3 dpf. The numbers of embryos used in each staining experiment and of those showed the presented patterns are labelled as denominators and numerators, respectively. **b** Normal vascular development as indicated by confocal microscopy images of *flk1:EGFP*-labelled blood vessels in the trunk of the heterozygous and homozygous *setd2* mutants at 50 and 72 hpf. **c** RT-qPCR analysis of the expression levels of representative vasculogenic genes, whose mouse orthologs have been known to be up- and downregulated in *Setd2* knockout mice^19^. The analyzed *setd2* mutant zebrafish embryos were at 72 hpf. In **b**, **c**, the embryos were produced by a cross between heterozygous and homozygous zebrafish so that the WT could only be analyzed separately and therefore was not shown. **P < 0.01; n.s., not significant.

Formation of blood vessels is a critical event both in the evolution from simple to complex species^1^ and in the development from the earliest non-vascular embryo, which receives nutrition by diffusion, to the highly vascular organism capable of quickly redistributing nutrition throughout the body^49^. Consistent with this notion, blood vessel development is induced, and tightly regulated, by various nutrient stresses such as hypoxia and amino acid starvation. In mammals, Setd2 plays an important role in this process, and loss of Setd2 leads to embryonic lethal at embryonic day (E) 10.5–11.5 due to a failure of embryonic vascular remodeling^19^. In sharp contrast, the homozygous *setd2* mutant zebrafish can grow through the embryogenetic stage without apparent abnormalities; whole-mount in situ mRNA hybridization analysis with organ-specific markers indicated that their major organs, including liver (marked by *lfabp*), intestine (*ifabp*), thymus (*rag1*), heart (*cmlc2*), pancreas (*trypsin*), and pancreatic islet (*insulin*), are completely normal in the shape, size, location and developing stage (Fig. 2a) (analyses of the three mutant lines produced similar results; herein shown is E3fs1, which will be used as the default setd2 loss-of-function model in the following parts of the manuscript).

We then closely analyzed the blood vessel development in the *setd2*-null embryos. An important tool for studying zebrafish blood vessels is the transgenic line Tg(*flk1:EGFP*), in which the blood vessels are labelled with the enhanced green fluorescent protein (EGFP) driven by the vascular endothelial cell-specific *flk1* promoter^50^. Therefore, we crossed between this Tg(*flk1:EGFP*) line and our *setd2*-null mutant, and fluorescent microscope imaging of the EGFP-expressing embryos at different stages showed that the loss of Setd2 did not impair the vascular development in zebrafish (Fig. 2b). To further clarify this issue at molecular level, we performed RT-qPCR to analyze the vasculogenic genes whose mouse orthologues had been known to be up- or downregulated in the *Setd2* knockout mice^19^. The results showed that none of these genes were altered by loss of Setd2 in zebrafish (Fig. 2c), thus further supporting that, unlike mouse, zebrafish does not require Setd2 function in their vascular development.

### Nutrition-dependent, reversable smaller body size of *setd2*-null zebrafish

**Fig. 3:**
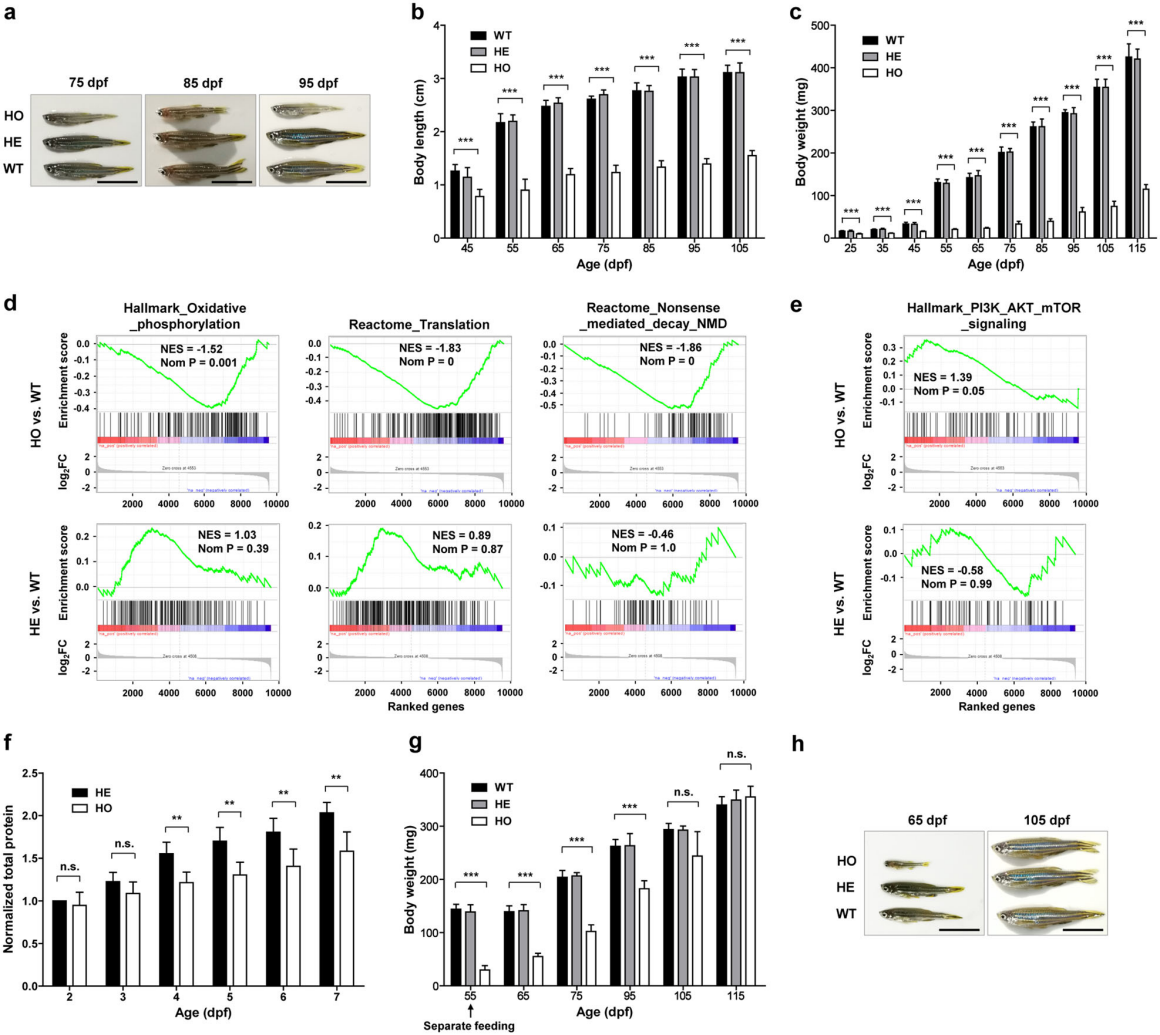
Nutrition-dependent, reversable smaller body size of the *setd2*-null zebrafish. **a** Photographs of representative wild-type and *setd2* mutant zebrafish growing in regular culture condition. **b**, **c** Quantification and statistical analysis of body length and body weight of the wild-type and *setd2* mutant zebrafish of indicated ages. **d** Gene set enrichment analysis (GSEA) of RNA-seq data of the embryos at 36 hpf, showing that the genes related to energy metabolism, protein translation, and NMD are depleted in the homozygous but not the heterozygous *setd2* mutant embryos, compared with the wild-type. **e** Activation of the PI3K-AKT-mTOR signaling pathway in the homozygous *setd2* mutant embryos. **f** Quantification of total protein levels of individual dechorionated and deyolked embryos at early ages. **g** Rescue of the small body size phenotype by a separate feeding strategy started at 55 hpf. **h** Photographs of representative wild-type and *setd2* mutant zebrafish growing in the separate feeding condition as in **g**. In **a**, **h**, scale bars = 1 cm. In **b**, **c**, **f** and **g**, data are showed as means ± SD; two-tailed *t*-test; ***P < 0.001; **P < 0.01; n.s., not significant.

While the *setd2* mutant zebrafish embryos have normal blood vessel development and organogenesis, the adult homozygous growing in the regular culture condition were found to have notably shorter body length, but without any developmental retardation at specific stage, compared with the heterozygous and wild-type siblings (Fig. 3a). This difference could be significantly measurable after 45 day post fertilization (dpf) (Fig. 3b) and, accordingly, the body weight of the homozygous were also lower than the heterozygous and wild-type siblings, which was started to be significantly measurable by an analytical balance as early as 25 dpf (Fig. 3c). Consistent with previous reports that the domesticated zebrafish strains (e.g., the herein used Tübingen strain) lack the natural sex-determination system^51^, and that their rapid growth can increase the proportion of female^52^, we found that the homozygous *setd2* mutants had much smaller number of female under regular culture condition. Nonetheless, there was no obvious difference in food intaking or swimming behaviors between the mutants and the wild-type, as indicated by comparable frequencies of bite, areas of swimming, and involuntary tail movements, although these measurements could not exclude other ethological differences such as ability or willingness of competition, which may play a role in these phenotypes.

To understand the underlying mechanism of how the *setd2* deficiency causes this small body size phenotype, we performed RNA-seq analysis of the homozygous, heterozygous and wild-type littermate embryos at 36 hpf. The age of 36 hpf was chosen because at such an early stage the mutant phenotype has not emerged, and the nutrition supplied by the yolk is sufficient to support the growth of the embryo, so that the altered molecular mechanism at this stage may function as a cause rather than a consequence of the phenotype. Indeed, our gene set enrichment analysis (GSEA) of the RNA-seq data revealed that the genes associated with oxidative phosphorylation and protein translation are significantly depleted in the homozygous, but not the heterozygous, *setd2* mutant embryos, compared with the wild-type (Fig. 3d, left and middle). Meanwhile, as it has been shown that inhibition of NMD acts as an important compensatory response to amino acid starvation^53^, we indeed found that the NMD-associated genes were also significantly downregulated in the *setd2*-null embryos (Fig. 3d, right), which, as reported^53^, could promote amino acid homeostasis by selectively retaining the transcripts with amino acid metabolism functions. Thus, these results suggest that the decreased activities of energy metabolism and protein synthesis have already been evident in the *setd2*-null embryos at early stage. Furthermore, we also found that the PI3K/AKT/mTOR signaling pathway was considerably activated in the *setd2*-null embryos (Fig. 3e), which possibly could improve the survival and growth of the *setd2*-null embryos that were suffering insufficient energy metabolism and protein synthesis, likely through the capacity of the PI3K/AKT/mTOR axis for sensing and responding to these stresses^54^. Interestingly, a direct cross-species comparison of the gene expression regulated by loss of *Set2*/*Setd2* showed similar patterns in the yeasts under nutrient stress and the zebrafish and mouse embryos (Supplementary Fig. S4 and data not shown), which suggests an evolutionary conservation of these mechanisms.

Based on these possible mechanisms, we made two hypotheses: (i) a reduction of protein synthesis could be an early symptom that associates with the smaller body size of the *setd2*-null embryos; and (ii) an increase of nutrition supply may rescue, at least to a certain degree, this mutant phenotype. To test these hypotheses, we first quantified the total protein levels of individual embryos at different stages and compared between the *setd2*-null embryos and the siblings. As a result, it was as early as 4 dpf when the significantly different total protein levels of individual embryos were detectable (Fig. 3f), which suggests that the reduced protein synthesis indeed occurs very early, although it seems not severely affect the embryogenesis. We then performed a separate culturing and feeding strategy to increase nutrition and to prevent competition between the embryos. The results showed that this strategy largely rescued the small body size phenotype of the *setd2*-null embryos. Furthermore, a decrease of rearing density in mixed culture could also diminish the body size difference of the homozygous *setd2* mutants compared with the wild-type and the heterozygous siblings (Supplementary Fig. S5). Meanwhile, the proportion of female of the homozygous *setd2* mutants was also increased in these conditions. To explore the mechanism underlying this rescue effect, we performed RNA-seq analysis of the livers of the rescued and the control zebrafish. The results revealed that the genes associated with metabolism and protein synthesis were upregulated (Supplemental Fig. 6 and data not shown), which suggests an overactivation of these pathways in the *setd2*-null zebrafish that received increased nutrition. These results may explain why the small body size phenotype can be rescued in these conditions. Notably, the liver was chosen for this analysis because it provides relatively homogenous tissue and is easily obtainable. However, the upregulation of the metabolism- and protein synthesis-related genes is unlikely restricted to liver but possibly occurs in many tissue/cell types. Lastly, It is also notable that, even though the separate culture was applied at a late timepoint (e.g., 55 dpf) when the mutant phenotype had already been established, this phenotype could still be rescued (Fig. 3g, h). Taken together, these results strongly suggest that the small body size phenotype of the *setd2*-null zebrafish is likely associated with metabolic mechanisms, and that this phenotype is reversable and dependent on the nutrition supply.

### *setd2*-null zebrafish are fertile in both male and female

**Fig. 4:**
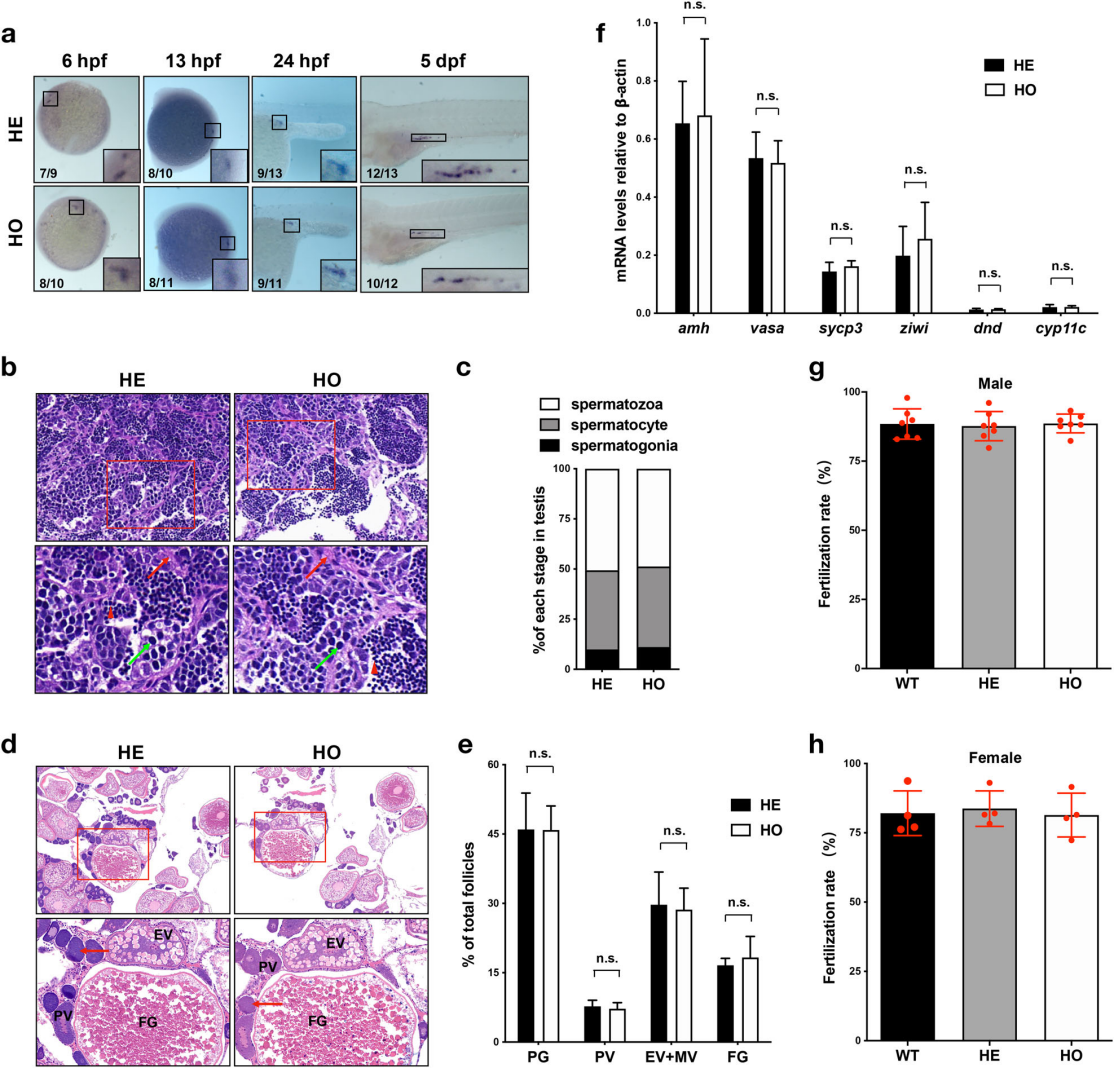
Normal germ cell development and fertilization of the male and female *setd2*-null zebrafish. **a** Whole mount in situ hybridization with *vasa* showing the PGCs in the homozygous (HO) and heterozygous (HE) *setd2* mutants at indicated stages. **b** H&E staining of the testes of adult *setd2* mutant zebrafish at 3.5 month-post-fertilization (mpf). The boxed areas in the upper panels are magnified in the lower ones. Red and green arrows and red arrowheads denote spermatogonia, spermatocyte, and spermatozoa, respectively. **c** Quantification of the indicated cell types showing normal percentages of the spermatogonia, spermatocyte and spermatozoa in the testes of the *setd2*-null zebrafish. **d** H&E staining of the ovaries of adult *setd2* mutant zebrafish at 3.5 mpf. The boxed areas in the upper panels are magnified in the lower ones. Red arrows denote the oocytes at primary growth stage (PG). PV, previtellogenic stage; EV, early vitellogenic stage; FG, full-grown stage. **e** Quantification of the indicated developmental stages, showing their normal pecentages in the total follicles. **f** RT-qPCR analysis of the germ cell-specific genes in the adult testes of *setd2* mutant zebrafish. **g**, **h** The ratio of successful fertilization by *setd2*-null male (sperm) and female (oocyte) with wild-type oocyte and sperm, respectively, in the in vitro fertilization assay. In **e**–**h**, data are showed as means ± SD. n.s., not significant.

We then investigated the reproductive functions of the *setd2*-null zebrafish. In contrast to the previous findings that the mouse *Setd2* is required for sperm and oocyte development^38,39,55^, and that the germ cell-specific *Setd2* knockout mice are sterile^38,39^, we surprisingly observed that both male and female homozygous *setd2*-null zebrafish could produce a number of progenies, suggesting that their reproductive functions should remain intact. To clarify this issue closely, we examined the reproductive processes of the *setd2*-null zebrafish ranging from germ cell development to fertilization. We first analyzed the primordial germ cells (PGCs), the precursors of both sperm and oocyte, by whole mount in situ mRNA hybridization with the germ cell-specific marker *vasa* (also known as *ddx4*)^56,57^. The results showed that the formation, migration, and number of PGCs in the homozygous *setd2*-null zebrafish were normal, as compared with those in the sibling controls (Fig. 4a). We then performed histological analysis of their testes and ovaries. Unlike the spermiogenic arrest caused by *Setd2* deficiency in mice^38^, our H&E staining of the testes of the adult *setd2*-null zebrafish showed that the lobular cavities were filled with morphologically normal mature sperms, which aligned tightly and in proper order with their precursor cells (Fig. 4b). Quantification of the different cell types showed normal percentages of the spermatogonia, spermatocytes and spermatozoa in the testes (Fig. 4c). Meanwhile, H&E staining of the ovaries of the *setd2*-null zebrafish also showed normal morphology and percentages of the oocytes at different stages, compared with the sibling controls (Fig. 4d, e). Furthermore, given that some germ cell-specific genes have been shown to be expressed in specific stages of spermatogenesis, we performed RT-qPCR analysis of several of these genes, including *amh* (Sertoli cells)^58^, *ziwi* (also known as *piwil1*; type A spermatogonia)^59^, *sycp3* (spermatocytes)^60^, *cyp11c1* (Leydig cells)^61,62^, as well as the germ cell markers *vasa*^56,57^ and *dnd*^63^, in the adult testes. The results showed that all these genes were expressed normally in the *setd2*-null zebrafish compared with the sibling controls (Fig. 4f). Lastly, to determine whether the *setd2* loss could affect the function of the sperms and oocytes, the homozygous and heterozygous *setd2* mutant, as well as the wild-type, sperms and oocytes were subjected to in vitro fertilization with wild-type oocytes and sperms, respectively, and the successful fertilization rate was indicated by formation of blastodisc. As a result, for both sperms and oocytes, the groups of different genotypes showed similar fertilization rates (Fig. 4g, h), thus suggesting that the setd2-null sperms and oocytes are fully functional in the fertilization.

### Maternal *setd2* regulates survival rate of zebrafish embryos

**Fig. 5:**
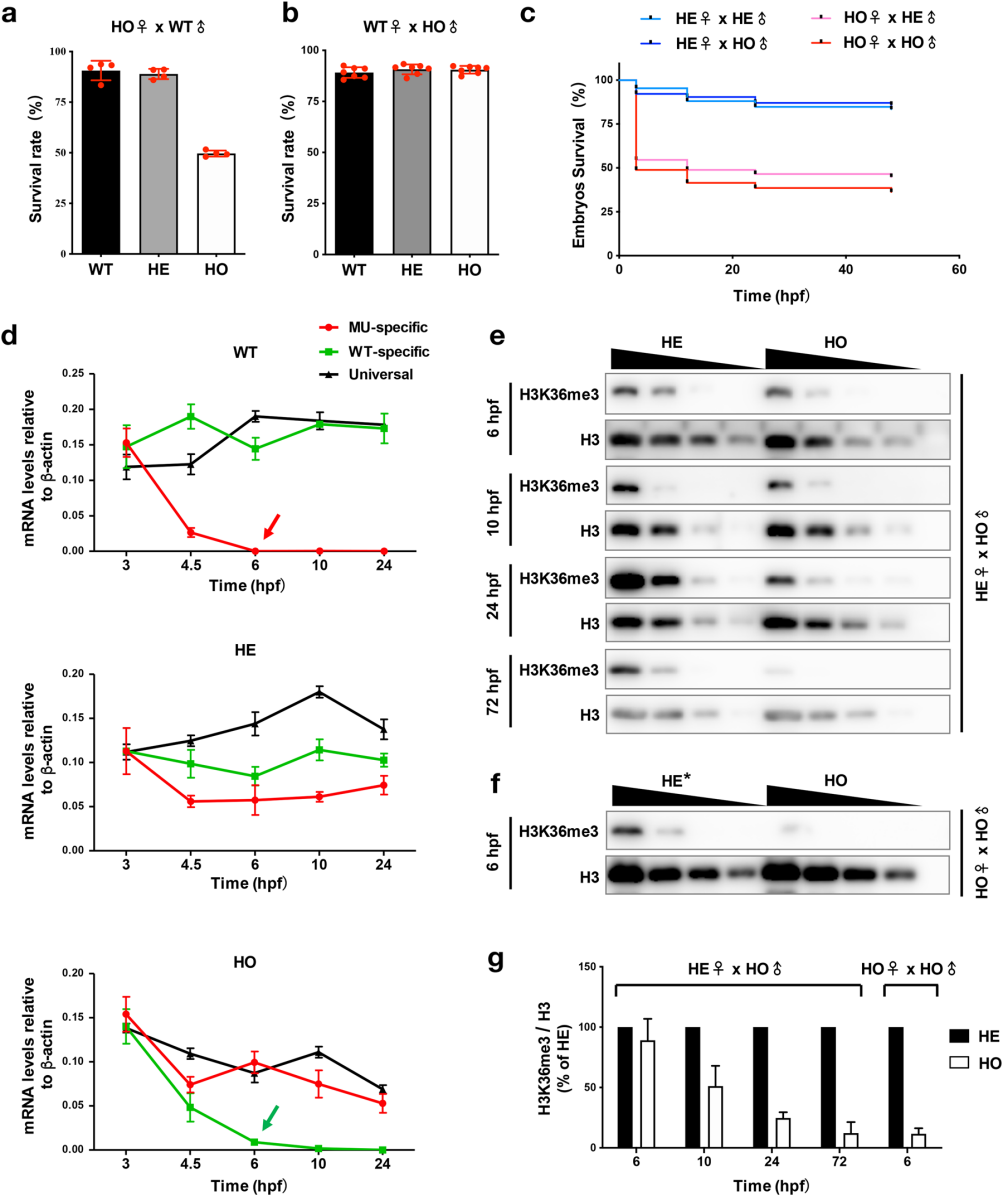
Maternal *setd2* regulates survival rate of zebrafish embryos. **a** Decrease of survival rate of the embryos produced by *setd2*-null (homozygous setd2 mutant) female and wild-type male zebrafish. **b** Normal survival rate of the embryos produced by *setd2*-null male and wild-type female zebrafish. **c** A direct comparison among the four mating relationships between the homozygous and heterozygous parents. Note that only the *setd2*-null female could determine the survival rate, regardless of the mating patterners. **d** Allele-specific RT-qPCR analysis of the wild-type and mutant *setd2* mRNAs along the development of the embryos of indicated genotypes. The red and green arrows denote the degradation of maternally deposited, mutant and wild-type alleles of *setd2* mRNAs in the wild-type and homozygous *setd2* mutant embryos, respectively. **e** Immunoblot analysis of H3K36me3 levels in the heterozygous and homozygous *setd2* mutant embryos that carry maternal *setd2*. Blots of histone H3 were used as loading controls. Each sample was loaded in 2-fold serial dilution. Note the gradual decrease of H3K36me3 along the 72 h of development. **f** Immunoblot analysis of H3K36me3 in the homozygous *setd2* mutant embryos in the absence of maternal *setd2*. Note that the control heterozygous embryos were impossible to be the littermate, therefore being labelled as “HE*”, because no heterozygous progeny could be produced by homozygous parents. Instead, the control heterozygous embryos were produced by crossing between homozygous male and wild-type female, and their H3K36me3 levels had been confirmed to be comparable with the “HE” use in **e**. **g** Quantification of the H3K36me3 levels in the indicated embryos based on the immunoblot results as shown in **e**, **f**.

We then examined whether the second generation of *setd2*-null zebrafish has any defect in their development. Interestingly, although a large number of the second generation of progenies could be produced by the *setd2*-null parents, we observed that, if the oocytes were derived from a homozygous *setd2* mutant female, the fertilized embryos (i.e., the second generation) would have a decreased survival rate, which was regardless of the genotypes of the embryos as homozygous or heterozygous, even if the paternal genome was wild-type (Fig. 5a). In contrast, this decrease of survival rate was not seen in the mating between homozygous *setd2* mutant sperms and wild-type oocytes (Fig. 5b). This finding was further validated by a comparison of four mating relationships between the heterozygous and homozygous *setd2* mutants, which confirmed that, for example, the mating between homozygous oocytes and heterozygous sperms (HO♀ × HE♂), but not the opposite mating relationship (HE♀ × HO♂), led to the decreased survival rate (Fig. 5c).

A possible explanation of these observations is that *setd2* is maternally deposited in the oocytes, which must play an important role in supporting a high survival rate of the fertilized embryos. Indeed, our previous genome-wide developmental expression profiling studies have identified *setd2* as one of the maternal histone methyltransferases in zebrafish^64^. As the herein generated *setd2* mutant zebrafish carry maternal *setd2* mRNAs that are distinguishable from the zygotic ones by allele-specific RT-qPCR (i.e., the wild-type embryos carry maternally deposited mutant *setd2* mRNA, and the homozygous *setd2* mutant embryos carry wild-type mRNA), they provide an ideal model to further verify the deposition, function, and degradation of the maternal *setd2* mRNAs during embryonic development. To this end, we designed PCR primers that could specifically detect the wild-type or the mutant *setd2* mRNAs (Supplementary Fig. S7) and analyzed the embryos at different stages. The results showed that the maternal *setd2* mRNA, both wild-type and mutant, were indeed present in the embryos and were significantly degraded by 6 hpf (Fig. 5d). Furthermore, immunoblot analysis of H3K36me3 levels in the *setd2* mutant embryos also showed a trend toward decrease of H3K36me3 in the homozygous embryos that carried maternal *setd2* mRNA and protein, and this decrease was much slower than the *setd2* mRNA degradation (Fig. 5e). In contrast, in the embryos that were produced by homozygous parents and thus carried no maternal *setd2* mRNA/protein, the H3K36me3 was quickly decreased (Fig. 5f). Therefore, this comparison suggests that, compared with the actively degraded *setd2* mRNA, it takes more time to degrade the maternally deposited or maternal mRNA-translated Setd2 protein. Taken together, these results are strongly supportive of the existence and the potential function of the maternal *setd2* in the embryonic development.

### Loss of maternal *setd2* causes a developmental delay at maternal-to-zygotic transition

**Fig. 6:**
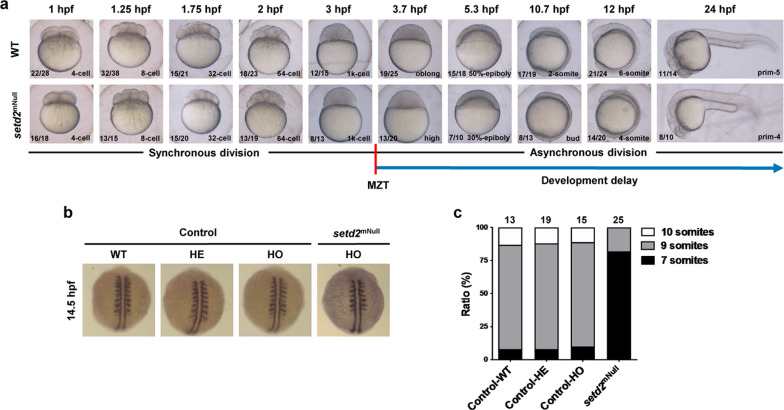
Loss of maternal *setd2* causes a developmental delay at MZT. **a** A comparison between the wild-type (WT) and the maternal *setd2*-depleted (*setd2*^mNull^) embryos of indicated ages. The actual developing timepoints (in hpf) of the embryos are written on top, whereas the morphological stages are labelled in the lower right corners of the images. The red vertical bar denotes the MZT, which occurred after 3 hpf or 1k-cell stage, and the blue arrow denotes the developmental delay after MZT, as indicated by asynchronous division of the embryos. The numbers of embryos analyzed at each time point and of those showed the presented developmental stage are labelled as denominators and numerators, respectively. **b** Different somite numbers of the *setd2*^mNull^ embryos compared with control embryos carrying maternal *setd2*. The control embryos with different genotypes were produced by self-crossing of heterozygous *setd2* mutant zebrafish. The somites were labelled by whole mount in situ mRNA hybridization with *myod*. **c** Ratios of the *setd2*^mNull^ and control embryos having indicated numbers of somites. The numbers labelled on the top denote the numbers of embryos analyzed for each group in **b**, **c**.

Previous mouse studies have shown that the maternal *Setd2* is absolutely required for proper zygotic genome activation (ZGA), and maternal depletion of *Setd2* causes an arrest of development at 1-cell stage when the ZGA is initiating^39^. In zebrafish, ZGA occurs when the embryo generates ~1000 (1k) cells at 3 hpf, which is called maternal-to-zygotic transition (MZT). The apparently normal embryogenesis of many embryos that lack maternal *setd2* (i.e., the second-generation progenies produced by *setd2*-null female; herein written as *setd2*^mNull^) indicated that the loss of maternal *setd2* in zebrafish does not cause a complete arrest at MZT. However, we found that, while the *setd2*^mNull^ embryos could keep the pace of the wild-type embryos to develop to the 1k-cell stage at 3 hpf, they exhibited a short delay afterward (Fig. 6a). In particular, when the wild-type embryos reached the oblong stage at 3.7 hpf, the *setd2*^mNull^ embryos still remained at the high stage (Fig. 6a) and, subsequently, the *setd2*^mNull^ embryos consistently showed a significant delay equivalent to 0.5–1 h of normal development^65^, compared with the wild-type embryos (Fig. 6a). These results suggest that this developmental delay of the *setd2*^mNull^ embryos is tightly associated with MZT. Notably, a similar phenotype has been observed in the zebrafish embryos that lack maternal Ythdf2, and m^6^A-binding protein^66^, thus suggesting a potential link between Setd2/H3K36me3 and m^6^A mRNA modification in the regulation of MZT.

To further confirm this phenotype, and to exclude the possibility that the loss of zygotic *setd2* (i.e., in the homozygous *setd2* mutant) may also contribute to this developmental delay, we compared the *setd2*^mNull^ embryos and the embryos produced by self-crossing of the heterozygous setd2 mutant; we also performed whole-mount in situ hybridization analysis of *myod*, which is specifically expressed in the somites and thus can be used to define developmental stages^67^. The results showed that, in the presence of maternal setd2, the wild-type, heterozygous and homozygous *setd2* mutant embryos at 14.5 hpf could normally reach 9-somite stage, whereas most of the *setd2*^mNull^ embryos remained at 7-somite stage (Fig. 6b, c). Notably, these statistics (i.e., almost all *setd2*^mNull^ embryos undergoing developmental delay at MZT) are different from the above-described decreased survival rate (~50%), suggesting that the MZT-associated developmental delay and the decrease of survival rate are distinct phenotypes. Taken together, these results identify a specific role of the maternal *setd2* in regulation of MZT, which is independent of whether *setd2* is present in or absent from the zygotic genome.

## Discussion

**Fig. 7:**
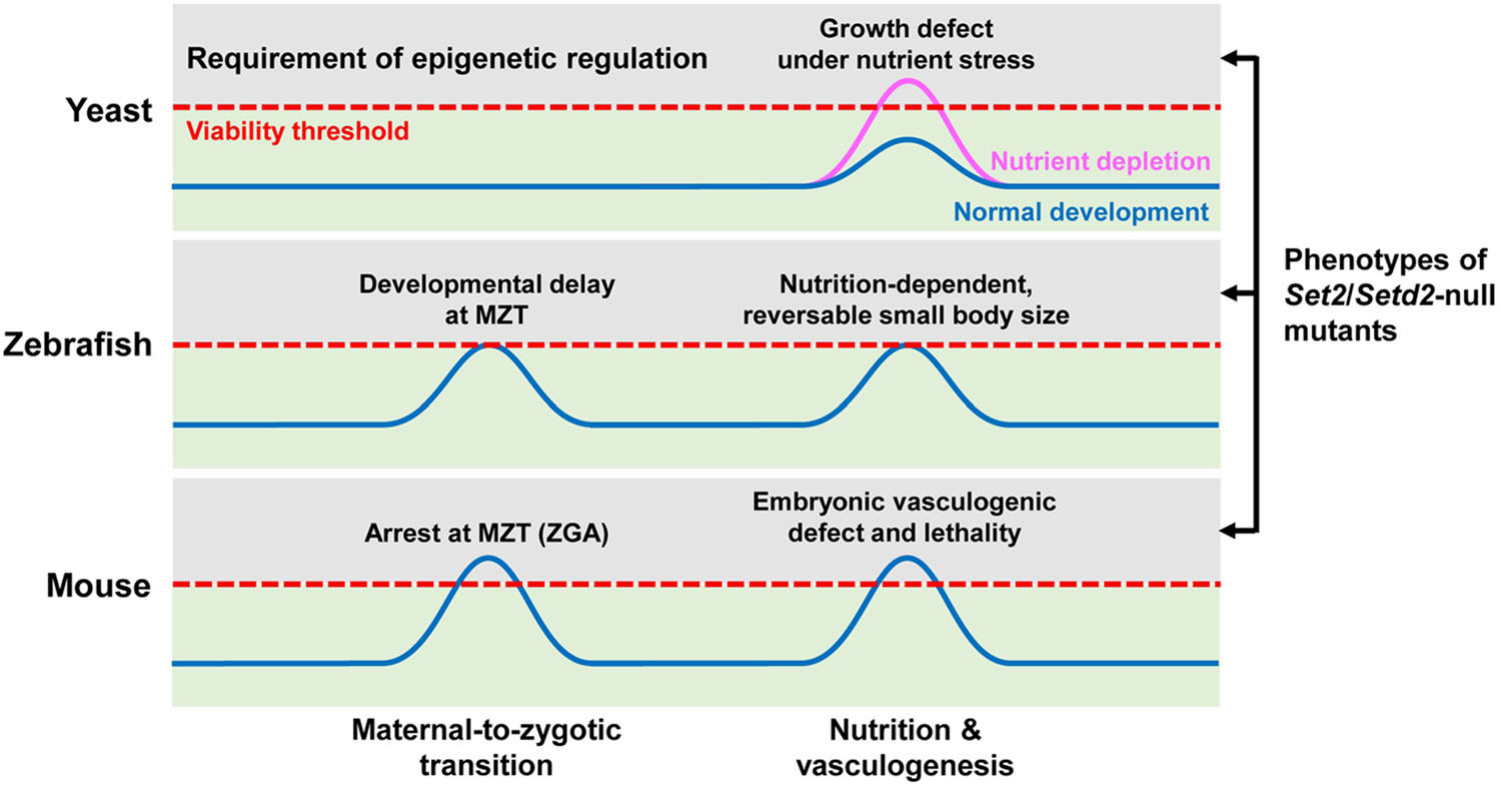
A working model to explain the apparently different, but actually paralleled, phenotypes of the *Set2*/*Setd2*-null yeast, zebrafish and mouse. The blue curves denote the levels of stresses that the organisms experience during normal development. The pink curve for yeast denotes an increased level of stress suffered by the yeast growing in nutrient depletion condition. The peaks of these curves are aligned vertically into two groups because they likely share conserved mechanisms in both nutrition and reproduction regulations. The red dashed lines denote the viability thresholds of different species when *Set2*/*Setd2* is lost, and, therefore, the gray regions above the thresholds mean that only in this region where the *Set2*/*Setd2*-mediated epigenetic regulation is required. Therefore, if the peaks of the blue/pink curves reach above the red dashed lines, it means that the levels of stress are too high to be tolerated by the *set2*/*setd2*-null organism, thereby leading to lethality. In the cases of zebrafish, it is proposed that, as the stresses just reach (but not above) the viability threshold, they cause tolerable and apparently minor (but clearly significant) defects in the *setd2*-null zebrafish, thus explaining the non-requirement of *setd2* in zebrafish development.

In this study, to gain a better understanding of the physiological function of Set2/Setd2 and H3K36me3 in an evolutionary context, we generated the *setd2* mutant zebrafish and analyzed their phenotypes in a comparison with those of the yeast and mouse models. Given the high conservation of genetic and epigenetic regulatory mechanisms in vertebrate animals, it is surprising that the *setd2*-null zebrafish seem to have dramatically different phenotypes, especially in their viability and fertility, compared with the *Setd2*-null mice. However, our close studies reveal some interesting phenotypes in zebrafish, including the nutrition-dependent, reversable small body size and the developmental delay at MZT, which could be mechanistically connected with the known phenotypes in yeast and mice. These findings suggest that, despite the apparently different phenotypes in these species, the functions and mechanisms of Set2/Setd2 and H3K36me3 are actually conserved through the evolution of eukaryotes, even though they sometimes are non-essential for normal growth. Further, the seemingly different requirements of Set2/Setd2 and H3K36me3 in the development and reproduction of these species is likely attributed to the different levels of stresses associated with these functions that can be tolerated by the organism. Therefore, based on these studies, we propose a working model (Fig. 7) to explain the apparently different, but actually paralleled, phenotypes of the *Set2*/*Setd2*-null yeast, zebrafish and mouse.

First, by comparing the three species, this model explains why the different phenotypes can be matched in parallel (Fig. 7, aligned peaks of the curves). In yeast, a phenotype of the *Set2*-null mutant is the growth defect under nutrient depletion but otherwise developing normally (in regular culture condition)^36,37^, suggesting a stress-related function of Set2. In the *setd2*-null zebrafish, the herein identified reversable phenotype of small body size also represents a growth problem and it is entirely dependent on nutrition supply, thus highly reminiscent of the yeast phenotype. Furthermore, our gene expression analysis reveals a decrease of the genes associated with energy metabolism and protein synthesis in the *setd2*-null embryos at early stages, thus providing a possible mechanism underlying this phenotype. Interestingly, these mechanisms seem conserved in yeast and vertebrates. In the *Setd2*-null mouse, although the phenotype in vasculogenesis^19^ seems unique and is not seen in zebrafish, it is probably attributed to the incompetence of the mutant mouse embryos in coping with the intrinsic nutrient stress. Indeed, as reported in our previous study^19^, a small body size phenotype actually is also observed in the *Setd2*-null mouse embryos at E8.5, and this phenotype is prior to the vasculogenic defect (at E10.5). Furthermore, recent studies showed that nutrient stress, such as amino acid restriction, can act as a proangiogenic trigger in mice^68^. Thus, the nutrient stress, small body size and vasculogenic defect can be mechanistically interconnected, suggesting the function and mechanism of Set2/Setd2 in regulating these phenotypes are evolutionarily conserved. Similarly, another group of paralleled phenotypes are that both *Setd2*-null zebrafish and mouse embryos show defects at the stage of MZT (or ZGA), which also suggest evolutionarily conserved functions and mechanisms of Setd2 in the regulation of reproduction.

Second, this model explains why the requirements of Set2/Setd2 for the viability of these species are different. Based on the present and previous studies, we use the concept of “viability threshold”, which represents the ability of an organism to tolerate certain factors such as stresses, to address this issue (Fig. 7, red dashed lines). While sharing many evolutionarily conserved molecular and cellular mechanisms, different species has distinct physiological structures and living environments, and therefore their viability thresholds can be widely variable^69^. For example, it has been known that the zebrafish embryos can obtain nutrition from the yolk by passive diffusion, so that they can tolerate higher degree of nutrient stress and have less dependence on the blood vessels to obtain and redistribute nutrition during early embryogenesis^70^. In contrast, the mouse embryos do not have a yolk as a source of nutrition, and they must rely on the development of a complex vascular system to obtain nutrition from the mother^69^. This difference may explain the apparently different phenotypes of *setd2*-null zebrafish and mouse embryos in the body size, blood vessel development and embryonic viability, which further supports that Setd2 and the related epigenetic regulatory mechanisms are important for the organisms to properly respond to nutrient stress. Another example is about the different phenotypes of the maternal *setd2*-depleted zebrafish and mouse embryos at the stage of MZT — while the loss of maternal Setd2 leads to a complete arrest of the mouse embryos, it seems to cause only a short delay of zebrafish embryos at this stage. It is conceivable that these phenotypes are regulated, at least partially, by conserved mechanisms of Setd2 in reprogramming the epigenome, as recently observed in mice^39^, and that the difference in the phenotypes is possibly related to some specific features of these species. In this regard, an important feature of zebrafish embryos is that the MZT is equivalent to 1000-cell stage, relative to mouse MZT at 2-cell stage^71^. Furthermore, epigenetic reprogramming of parental genomes in zebrafish also show some differences from that of mice^72–74^. Thus, although the detailed mechanism remains to be clarified, these morphological and mechanistic differences between zebrafish and mouse embryos may be directly relevant to the different requirements of maternal Setd2 for the embryos to complete MZT.

Lastly, by establishing the connection between Set2/Setd2 and the abilities of the organisms to cope with stresses, this model reemphasizes the important role of the epigenetic programs as a reserved source supporting the evolution of living beings from simplicity to complexity^2,75^. In the evolution, vasculogenesis and sexual reproduction represent two major advantages for multicellular organisms to obtain and redistribute nutrition and to maintain genetic stability and diversity^1^. It is interesting that Set2/Setd2 is involved into both processes. This is probably because the emergence of these processes had relied on the existing regulatory mechanisms capable of dealing with extra stresses, such as Set2/Setd2 and related epigenetic mechanisms. As such, the role of Set2/Setd2 should be changed from non-essential to essential for some specific developmental processes, as well as the viability. Also related to this notion, the epigenetic regulators (e.g., the homologues of Set2/Setd2) have been greatly expanded in the numbers, specificities, and regulatory mechanisms through eukaryotic evolution, and they play nonredundant roles in many aspects of development^64,75^. Taken together, these results and analysis suggest that the epigenetic regulatory system may serve as a buffer not only for coping with intrinsic and/or extrinsic stresses but also for the evolution of life from simplicity to complexity.

## Materials and methods

### Zebrafish strains

The zebrafish wild-type Tübingen strain (ZFIN ID: ZDB-GENO-990623-3) and the transgenic zebrafish line Tg(*flk1:EGFP*)^50^ (ZFIN ID: ZDB-ALT-050916-14) were used in this study. All zebrafish husbandry was achieved under standard conditions (temperature: 28.5 °C; pH: 7.0–8.0; light cycle: 12 h-on/12 h-off). The embryos were maintained in egg water (containing 60 μg/mL sea salt and 0.2% methylene blue) at 28.5 °C and 0.045% *N*-Phenylthiourea (PTU; Sigma-Aldrich) was performed to prevent pigmentation.

### Establishment of *setd2* mutant lines by the CRISPR/Cas9 system

The genomic sequence of zebrafish *setd2* gene was obtained from the National Center for Biotechnology Information. An online tool (ZiFit Targeter, http://zifit.partners.org/ZiFiT/Disclaimer.aspx) was used to design guide RNA (gRNA) target sites in exon 3 and exon 19 containing 20-base pair (bp) target sequences (E3: cgtcaaaatgtgtttctacc; E19: ggcagacacgtccagtgagc). The predicted sequence was cloned into p-T7-gRNA vector backbone to generate the guide RNA expression construct. The specific single-guide RNAs (sgRNAs) was produced by in vitro transcription with the mMESSAGE mMACHINE SP6 Transcription Kit (Thermo Fisher, AM1340). The sgRNA (10 ng/μL) and Cas9 protein (200 ng/mL) (EnGen Cas9 NLS; New England Biolabs, M0646T) were co-injected into zebrafish embryos at the one-cell stage. A dozen of injected embryos were subjected to DNA extraction and genotyping at 24 hpf. The rest of founders (F0) were raised to adulthood and the individuals carrying mutations were identified by genotyping on caudal fin with sequencing. The F0 founders bearing mosaic mutations at the target sites were then outcrossed with WT fish to produce F1 heterozygotes. The F1 heterozygotes harboring same frame-shift mutation were incrossed to generate F2 homozygous mutants.

### Protein quantification

To compare the total amount of embryos protein at different time points during the early development, every embryo was dechorionated and pipetted into standard protein RIPA lysis buffer for sonication. Then six embryos with same genotype were merged to perform BCA protein assay kit (Thermo Scientific, 23227). Three biological replicates were measured for each time point to determine the average protein amount.

### Immunoblot

Zebrafish embryos were deyolked as described previously^76^ and the proteins were extracted by RIPA buffer (Beyotime Biotechnology, P0013D) containing appropriate protease inhibitors (MedChemExpress, HY-K0010) with sonication. Boiled lysates were first separated with SDS-PAGE and transferred onto PVDF membranes. The separated proteins were immunoblotted with antibodies for H3K36me1 (Abcam, ab9049), H3K36me2 (Abcam, ab9048), H3K36me3 (Abcam, ab9050) and H3 (Cell Signaling Technology, 4499S).

### Live imaging of blood vessels

To observe the vasculogenesis of *setd2* mutants, the transgenic zebrafish line Tg(*flk1:EGFP*)^50^ was crossed with the *setd2* mutants. Live embryos were anesthetized with 0.03% Ethyl 3-aminobenzoate methanesulfonate salt (Sigma-Aldrich, A5040) and mounted in 1% low melting agarose (Sangon Biotech, A600015). Fluorescence images were captured with a scanning confocal microscope (Olympus, FV1000) processed with Image-Pro Plus 6.0 (Media Cybernetics).

### Whole-mount mRNA in situ hybridization

The PGCs were labelled with *vasa*; and the somites were labelled with *myod*. The major organs were labelled specific markers including *ifabp*, *lfabp*, *cmlc2*, *rag1*, *trypsin*, and *insulin*. Whole-mount mRNA in situ hybridization was performed as previously described^77,78^. Anti-digoxigenin Fab fragment antibodies conjugated to alkaline phosphatase (Roche, 1277073) were used for detection of the RNA probes, and this was followed by NBT/BCIP color reaction (Vector Laboratories, SK-5400).

### RNA isolation and quantitative reverse transcription PCR (RT-qPCR)

Total RNA was isolated with TRIzol reagent (Ambion, 15596018) according to the manufacturer’s instructions. The amount and purity of the RNA were determined by spectrophotometry and agarose gel electrophoresis. After treatment with RNase-free DNase, total RNA was reverse transcribed using random hexamers and oligo(dT) primers (Takara, RR037Q). qPCR was carried out on an ABI Prism 7900HT Sequence Detection System (Applied Biosystems) using the SYBR Green Real-Time PCR Master Mix kit (TOYOBO, QPK-201). The expression levels of target genes were normalized against the internal control *β-actin* (*actb1*) gene.

### RNA-seq and bioinformatic analysis

Total RNA was extracted from whole embryos at 36 hpf using TRIzol (Ambion, 15596018). Library construction with fragmented mRNA was performed with TruSeq stranded mRNA library prep kit (Illumina). Sequencing was carried out on Illumina HiSeq 2000 according to the manufacturer’s instructions. The deep-sequencing data were mapped to zebrafish genome version 10 (GRCz10) with Tophat and Cufflink (v2.2.1) was used to calculate the FPKM of each gene to represent their mRNA expression level. Gene Set Enrichment Analysis (GSEA) was performed to identify regulated signaling pathways and to interpret gene expression patterns globally.

### Histological analysis and morphometric measurement

After anesthetized with 0.03% Ethyl3-aminobenzoate methanesulfonate salt (Tricaine), the gonadal tissues were dissected fixed overnight in 4% paraformaldehyde (Servicebio, G1101-20) and then subjected to paraffin embedding. Serial sections (4 μm) were cut, deparaffinized, and stained with H&E. All genotypes zebrafish collected for morphometric analysis, including somatic length and body weight, were measured from juvenile to adult at a 10-day interval. Meanwhile, different genotype raising in same tank were isolated feeding at 55 dpf, as morphometric record continued.

## Supplementary information

Supplementary Figures

## Data Availability

RNA-seq data are accessible through the Gene Expression Omnibus (GEO) accession code GSE151238.

## Supplementary information

**Supplementary Information** accompanies the paper at (10.1038/s41421-020-00203-8).
