# Author Correction: *setd2* knockout zebrafish is viable and fertile: differential and developmental stress-related requirements for Setd2 and histone H3K36 trimethylation in different vertebrate animals

**DOI:** 10.1038/s41421-025-00854-5

**Published:** 2025-11-17

**Authors:** Dian-Jia Liu, Fan Zhang, Yi Chen, Yi Jin, Yuan-Liang Zhang, Shu-Bei Chen, Yin-Yin Xie, Qiu-Hua Huang, Wei-Li Zhao, Lan Wang, Peng-Fei Xu, Zhu Chen, Sai-Juan Chen, Bing Li, Aijun Zhang, Xiao-Jian Sun

**Affiliations:** 1 Shanghai Institute of Hematology, State Key Laboratory of Medical Genomics, National Research Center for Translational Medicine at Shanghai, Ruijin Hospital Affiliated to Shanghai Jiao Tong University School of Medicine, Shanghai, 200025 China; 2 School of Life Sciences & Biotechnology, Shanghai Jiao Tong University, Shanghai, 200240 China; 3 CAS Key Laboratory of Tissue Microenvironment and Tumor, Shanghai Institute of Nutrition and Health, Shanghai Institutes for Biological Sciences, University of Chinese Academy of Sciences, Chinese Academy of Sciences, Shanghai, 200031 China; 4 Division of Human Reproduction and Developmental Genetics, Women’s Hospital, and Institute of Genetics and Department of Genetics, Zhejiang University School of Medicine, Hangzhou, Zhejiang 310058 China; 5 Department of Biochemistry and Molecular Cell Biology, Shanghai Jiao Tong University School of Medicine, Shanghai, 200025 China; 6 Reproductive Medical Center, Ruijin Hospital Affiliated to Shanghai Jiao Tong University School of Medicine, Shanghai, 200025 China

Correction to: *Cell Discovery* 10.1038/s41421-020-00203-8, published online 20 October 2020

In this article, the authors identified that the affiliation for Dian-Jia Liu was incomplete. His correct affiliations should include the following affiliation:

School of Life Sciences & Biotechnology, Shanghai Jiao Tong University, Shanghai 200240, China.

We are sorry for the inconvenience.

