# Supplementary material for: setd2 knockout zebrafish is viable and fertile: differential and developmental stress-related requirements for Setd2 and histone H3K36 trimethylation in different vertebrate animals
Source: Cell Discov. 2020 Oct 20;6:72. doi: 10.1038/s41421-020-00203-8 (PMC7573620; doi:10.1038/s41421-020-00203-8)
Supplement: Supplementary file 1 — Supplementary Figures [file 41421_2020_203_MOESM1_ESM.pdf]

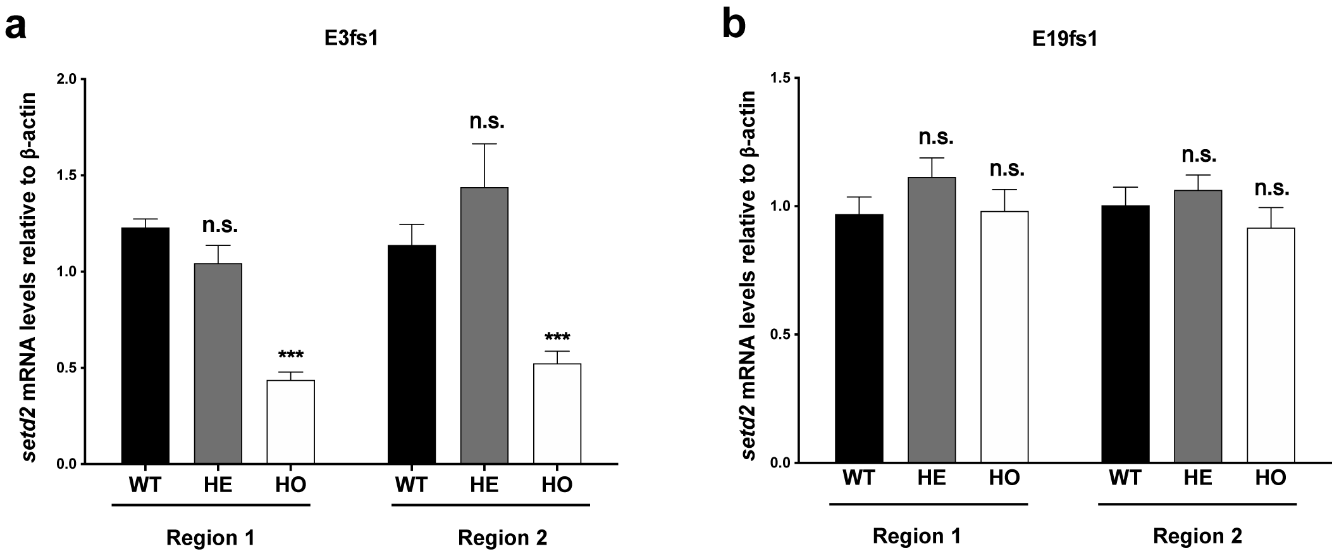

Supplementary Figure 1. RT-qPCR analysis of *setd2* mRNA levels in the E3fs1 and E19fs1 mutants at 3 dpf. (a) The *setd2* mRNA levels in the homozygous (HO), but not heterozygous (HE), E3fs1 mutants were dramatically decreased compared with the wild-type (WT), which was likely due to the nonsense-mediated mRNA decay (NMD) mechanism. **(b)** In contrast, the *setd2* mRNA levels in the E19fs1 mutants were not changed. In both (a and b), two regions in *setd2* mRNA were analyzed by qPCR and produeced the same results. Data are presented as mean ± SD of triplicate reactions. ***P < 0.001; n.s., not significant.

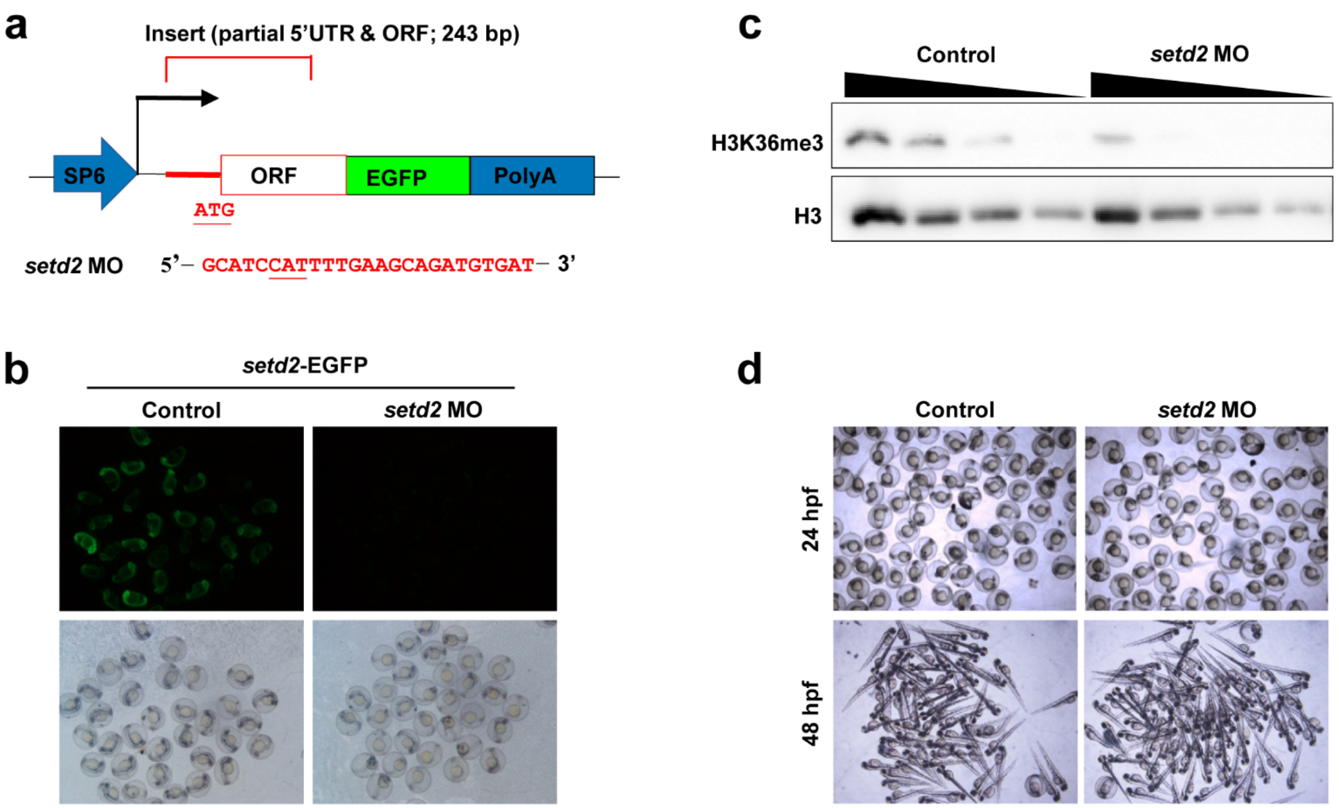

Supplementary Figure 2. Morpholino-mediated knockdown of *setd2* shows similar phenotypes as the *setd2* knockout regarding the decrease of H3K36me3 and the normal embryogenesis. (a) Targeting and validation strategy and sequence of the *setd2* morpholino oligo (MO), which is designed to inhibit protein translation. The partial 5'UTR and ORF of *setd2* mRNA containing the morpholino target sequence was fused in frame with EGFP followed by a polyA site. The start codon (ATG) in the targeted *setd2* ORF and its complementary sequence (CAT) in the morpholino is underlined. **(b)** Fluorescence microscopy (top) and bright field images (bottom) of representative embryos injected with the setd2-EGFP fusion mRNAs with or without *setd2* morpholino. Note that this morpholino can significantly inhibit the expression of EGFP, indicative of an effective knockdown. **(c)** Immunoblot analysis of H3K36me3 in the wild-type embryos and those injected with *setd2* morpholino. A blot of histone H3 was used as a loading control. Each sample was loaded in 2-fold serial dilution. **(d)** Normal embryonic development of the embryos injected with *setd2* morpholino.

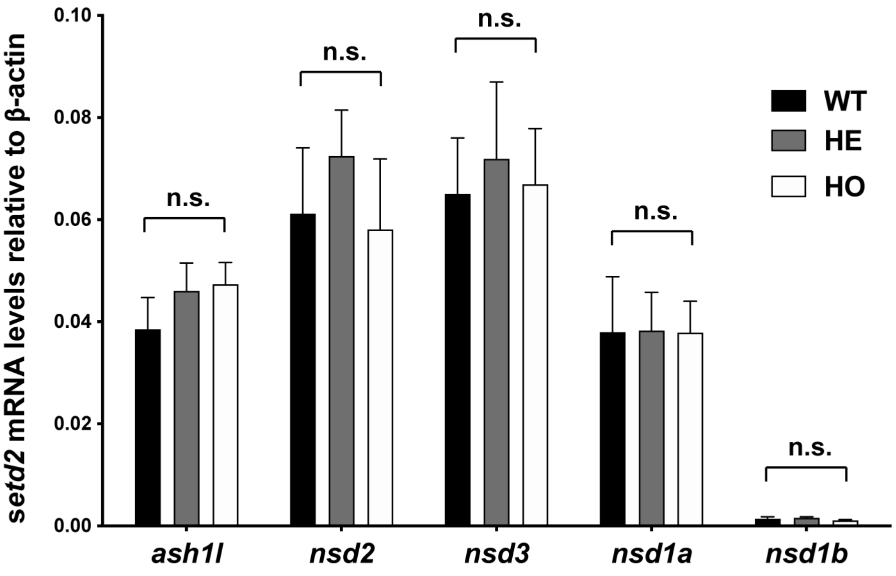

Supplementary Figure 3. RT-qPCR analysis of the mRNA levels of the closest homologous genes of *setd2* at 3 dpf. Note that none of these gene were changed in the *setd2* mutants compared with the wild-type, suggesting that the recently proposed genetic compensation mechanisms may not be applicable to the *setd2*-null model. Data are presented as mean ± SD of triplicate reactions. n.s., not significant.

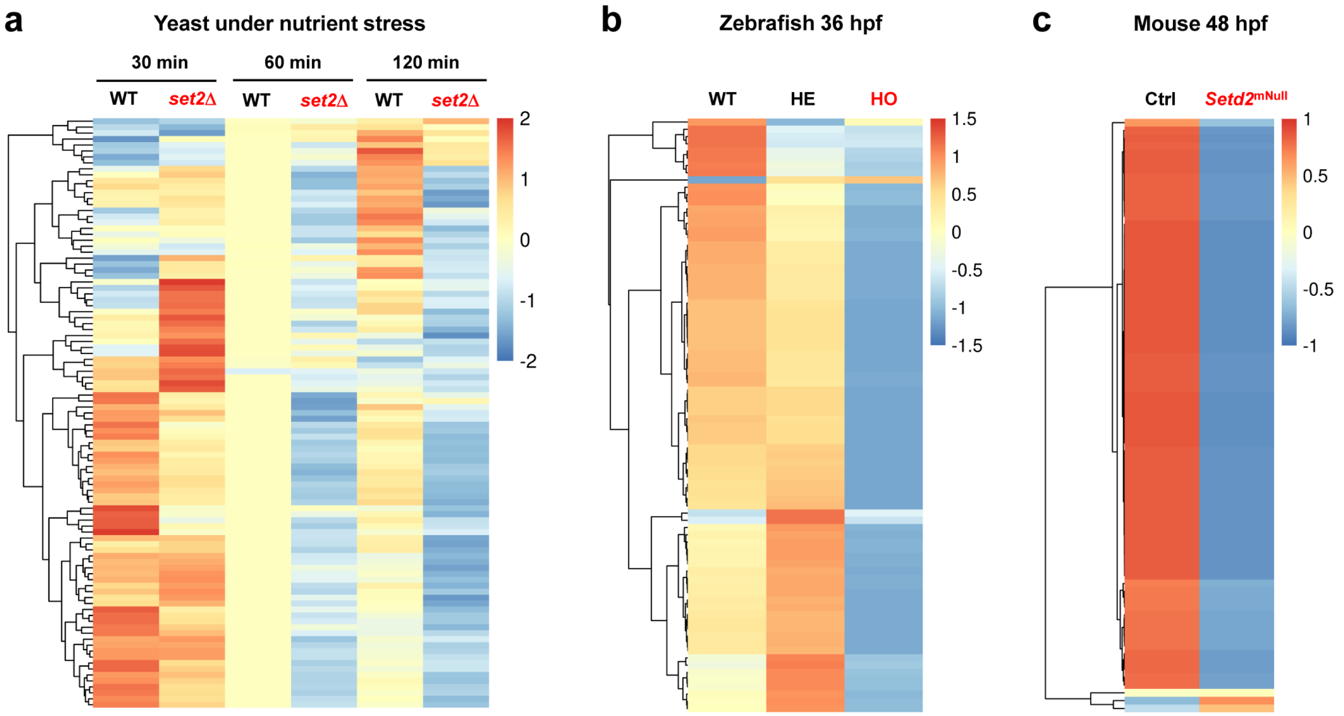

Supplementary Figure 4. Cross-species comparison of the regulation of ribosomal genes by loss of *Set2*/*Setd2*. The names of the *Set2*/*Setd2*-null mutants of yeast, zebrafish and mouse were in accordance with the original studies and were written in red. **(a)** Downregulation of ribosomal genes by loss of *set2* (*setd2*Δ) in the yeasts that are under nutrient stress for longer time (60 and 120 min) but not short time (30 min). RNA-seq data are referenced to McDaniel et al. 2017 (GEO acc #: GSE89265). **(b)** Downregulation of ribosomal genes in the homozygous *setd2* knockout zebrafish embryos at 36 hpf. **(c)** Downregulation of ribosomal genes in the maternal *Setd2* depleted (*Setd2*^{mNull}) mouse embryos at 48 hpf. RNA-seq data were referenced in Xu et al. 2019 (GEO acc #: GSE112832).

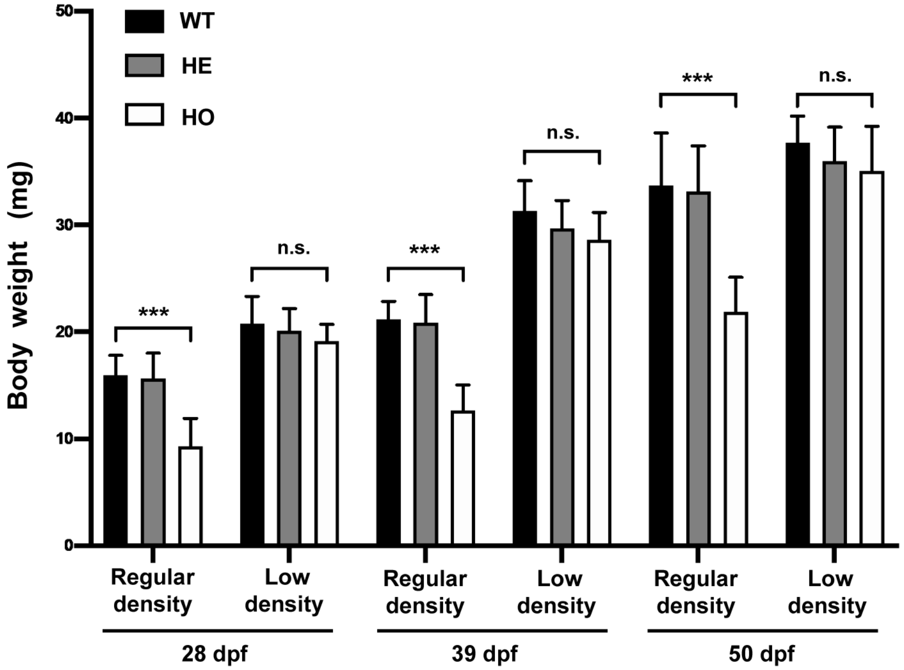

Supplementary Figure 5. A decrease of rearing density in mixed culture diminishes the body size difference of the homozygous *setd2* mutants compared with the wild-type and the heterozygous siblings. In this experiment, the regular and low rearing density are 35 and 4 zebrafish per 2.8-liter tank, respectively. Both groups were fed twice a day with excess food. Data are presented as mean ± SD of at least 9 zebrafish. ***$P < 0.001$; n.s., not significant.

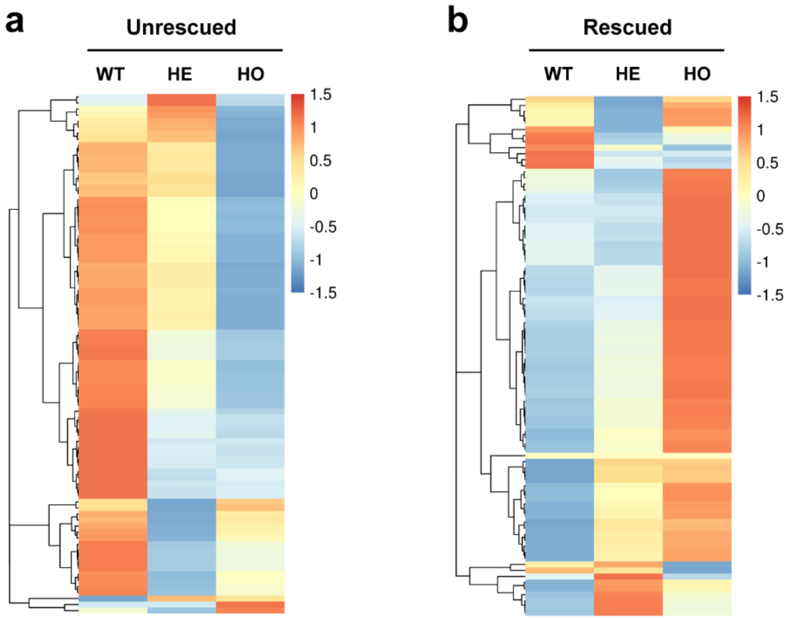

Supplementary Figure 6. Differential regulation of ribosomal genes in the livers of the nutrition-rescued and unrescued *setd2*-null zebrafish. (a) Downregulation of ribosomal genes in the liver of *setd2*-null zebrafish that are growing in normal culture condition and showing small body size phenotype. **(b)** Upregulation of ribosomal genes in the liver of *setd2*-null zebrafish whose body size has been rescued by separate feeding. The analyzed zebrafish were 3 months old and their separate feeding was started at 28 dpf.

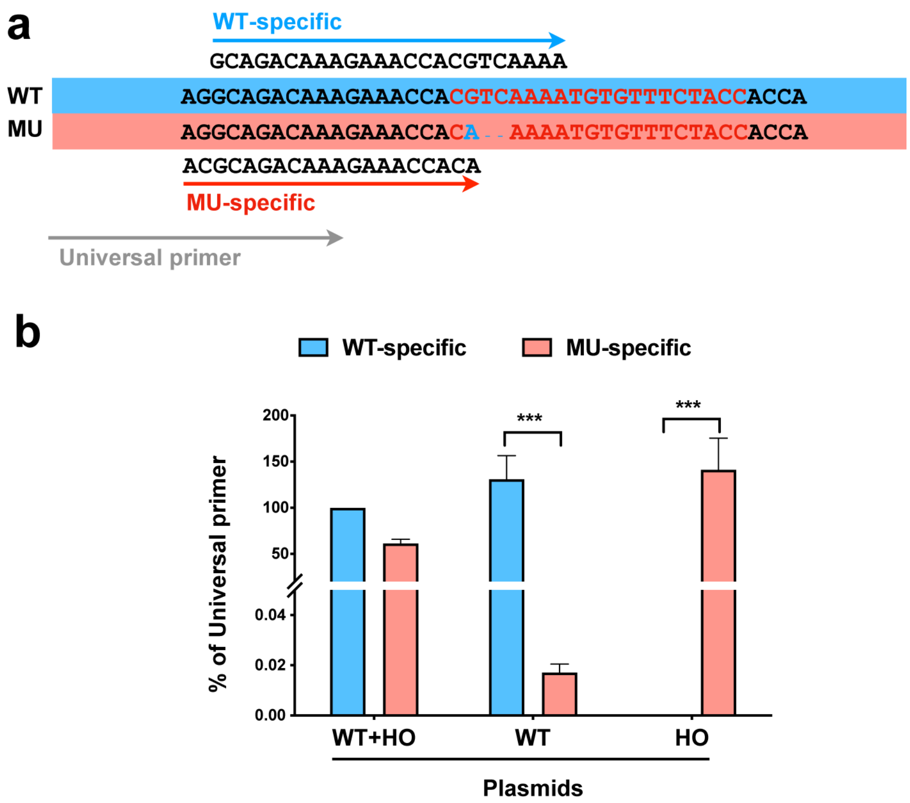

Supplementary Figure 7. Design and validation of the wild-type (WT) and mutant (MU)-specific primers for detecting the corresponding alleles effectively and specifically. (a) Design of the specific primers according to the WT and MU genomic sequences. A universal primer was also designed to amplify the both alleles. The downstream primers are not shown. **(b)** Efficiency and specificity of the primers as indicated by analyzing the plasmids containing the WT and HO alleles and their mix. The efficiencies were normalized to the universal primer. Data are presented as mean ± SD of triplicate reactions. ***$P < 0.001$.
